# Development of Competencies in Emergency Nursing: Comparison Between Self-Assessment and Tutor Evaluation Before and After a Training Intervention

**DOI:** 10.3390/nursrep14040259

**Published:** 2024-11-17

**Authors:** Marta Manero-Solanas, Noelia Navamuel-Castillo, Nieves López-Ibort, Ana Gascón-Catalán

**Affiliations:** 1 Hospital Universitario Miguel Servet, 50009 Zaragoza, Spain; nnavamuel@salud.aragon.es; 2 Instituto de Investigación Sanitaria de Aragón, 50009 Zaragoza, Spain; agascon@unizar.es; 3 Hospital Clínico Universitario Lozano Blesa, 50009 Zaragoza, Spain; 4 Departamento de Fisiatría y Enfermería, Facultad de Ciencias de la Salud, Universidad de Zaragoza, 50009 Zaragoza, Spain

**Keywords:** nursing, mentoring, clinical nurse, competence, professional development, self-assessment, evaluation

## Abstract

Background/Objectives: Nursing competence encompasses the integration of knowledge, skills, and attitudes essential for comprehensive and safe patient care. This study aimed to compare self-assessment and tutor evaluation of nurses’ competencies in a hospital emergency department before and after a training intervention. Methods: A quasi-experimental design was employed, involving 63 newly hired nurses who participated in a mentorship program. The intervention included theoretical and practical sessions on critical care skills. Data were collected through self-assessment questionnaires and objective evaluations by tutors using validated rubrics. Results: The results indicated significant differences between self-assessment and tutor evaluations in pre- and post-intervention phases, particularly in competencies related to orotracheal intubation and fibrinolytic therapy for ischemic stroke. Post-intervention, discrepancies between self-assessment and tutor evaluations decreased, suggesting improved self-awareness and competence among participants. Conclusions: This study highlights the importance of combining self-assessment and external evaluation to ensure accurate competency assessment and effective educational interventions, ultimately enhancing the quality of patient care.

## 1. Introduction

Nursing competence is not only the technical ability to perform clinical procedures but also refers to the integration of knowledge, skills, and attitudes that enable comprehensive and safe patient care [1,2]. Clinical environments are complex and multifaceted, requiring nurses to possess a combination of theoretical knowledge and practical skills to make quick and effective decisions. The complex reasoning processes that nurses use to make clinical judgments involve observing, interpreting, responding, and reflecting [2]. This dynamism demands that nurses be flexible and adaptable, capable of adjusting their approaches and strategies in real time [3]. Therefore, the development of nursing competence in clinical settings is essential for providing quality care and ensuring patient safety [4].

Many authors [5,6,7] propose improvements for workforce integration and emphasize the importance of promoting the role of the clinical tutor. This role provides resources to newly hired staff, allowing them to grow professionally, gain autonomy, and develop clinical skills with confidence. In the context of hospital emergency services, where speed and accuracy in decision making are crucial, nursing competence becomes even more significant.

Mentorship programs in clinical settings aim to enhance nurses’ competencies to face the unique challenges of a high-pressure and constantly demanding environment [8]. Continuous education and professional development are essential components for maintaining and improving nurses’ competencies, which in turn positively impact the quality of patient care and clinical safety [9]. To ensure the effectiveness of interventions and identify areas for improvement, the evaluation of training programs is crucial [2,10].

The literature indicates that to achieve a comprehensive and objective evaluation, a combined approach that includes both self-assessment and external evaluation should be adopted [9,11,12,13]. According to Farra et al. (2015) [14], behavioral changes and skill acquisition resulting from learning cannot be adequately assessed without complementary evaluation methods and tools that measure both the learning process and the acquisition of psychomotor skills [11].

Self-assessment is the process by which nurses gather evidence about their own performance, compare it with desired practice goals and standards, and set goals to improve their competence [10,15,16]. Participation in the self-assessment process helps nurses identify their weaknesses and strengths, increase self-confidence, and take greater responsibility for their learning [11,15,17].

On the other hand, evaluation by a tutor, conducted through validated rubrics, provides an external and objective perspective on the level of competence achieved by nurses [10]. Rubrics are assessment tools that establish clear and specific criteria for measuring performance, facilitating consistent and fair evaluation. The importance of objective evaluation in competence development cannot be underestimated. A well-structured and objective evaluation not only provides an accurate measure of performance but also identifies specific areas that require attention and improvement. This is crucial for designing effective educational interventions that address nurses’ development needs [17]. Moreover, objective evaluation helps ensure that the competencies acquired are relevant and applicable in clinical practice, which in turn enhances the quality of patient care.

The combination of self-assessment and evaluation by a tutor not only enriches the evaluation process but also promotes a culture of self-reflection and continuous improvement among nurses. Self-assessment fosters autonomy and self-efficacy [12,15], while external evaluation ensures that competency standards remain high and align with professional and organizational expectations. This dual approach is particularly relevant in the context of nursing, where self-assessment has been shown to have positive effects on individuals’ ability to organize their own learning [16].

### 1.1. Rationale of This Study

Some studies [9,10,11,15,18,19,20] reflect concerns that what is measured may not accurately reflect reality, and they describe discrepancies between self-assessment and tutor evaluation results. This could be consistent with the learning stages described by Martin M. Broadwell (1969) [21] and his four levels of the learning ladder: unconscious incompetence, conscious incompetence, conscious competence, and unconscious competence. Therefore, it is important to analyze how nurses perceive their performance compared to the tutor and whether there are factors contributing to agreement/disagreement.

Evaluating the congruence between self-assessment and tutor evaluation is important to ensure that both assessments provide a coherent and accurate picture of nurses’ competency levels [1]. These studies have generally been conducted with nursing students [22], so this study with nurses in a real work environment will provide valuable insights into the accuracy of the evaluation methods used.

### 1.2. Aim

The aim of this study is to compare the results of objective evaluation by the tutor and self-assessment of nurses’ competencies in a hospital emergency department before and after a training intervention and determine whether there are significant similarities or differences between the two evaluations.

## 2. Materials and Methods

### 2.1. Design

A prospective quasi-experimental study was conducted with a group of newly recruited nurses in a hospital emergency department to compare self-assessment of competencies with external agent evaluation before and after a training and competency development intervention.

Measurements were taken from October 2023 to August 2024 at two different points in time. The first measurement was taken before the intervention, and the second was taken after the intervention, within a period not exceeding one month.

### 2.2. Setting

This study was carried out in the Emergency Department of a public hospital with 1198 beds. It belongs to the Spanish National Health System, specifically to the autonomous community of Aragón. This department handles approximately 400 emergencies daily and has a structural staff of 96 nurses, in addition to temporary, seasonal, and substitute contracts, resulting in a significantly high staff turnover. During the year 2023, a total of 92 newly recruited nurses were welcomed, and 51 nurses joined from January to September 2024.

### 2.3. Population

The study population consisted of newly recruited nurses in the hospital emergency department. As an inclusion criterion, they must voluntarily agree to participate in the mentoring and competency development program for newly recruited nurses and have a contract exceeding 45 days in the unit. The exclusion criteria include not signing the informed consent, not participating in the tutor training sessions, and not completing all self-assessment questionnaires before and after the intervention.

### 2.4. Sample Size

The participants were included through convenience sampling. All personnel meeting the criteria listed in the previous section were included, resulting in a total sample of 63 participants. Given the specific participation criteria and the target population of new nurses, we decided to invite all eligible individuals rather than employing random sampling. The size of the sample and the accessibility of all qualifying participants made convenience sampling the most suitable option for our study. By offering the program to the entire eligible group, we reduced potential biases. Additionally, the statistical power of the sample size in this study was 0.98, indicating a strong likelihood of detecting a true effect if it exists.

### 2.5. Competency Development Intervention

The intervention involved the implementation of a tutoring program for the competency development of newly recruited nurses in a hospital emergency department. Led by a nursing tutor, this program comprises two phases. The first phase is an orientation program lasting two months, during which the novice nurse receives sessions and training for their integration into the job. The second phase, lasting one month, is a competency development program for nurses caring for critically ill patients. A total of five competencies are developed by the tutor over four two-hour sessions, which include both theoretical and practical content relevant to each topic. These sessions utilize real devices that are available in the service, ensuring that the training is both applicable and hands on. This approach enhances the learning experience and prepares participants for practical application in their roles. These five competencies are addressed in theoretical–practical face-to-face sessions, which are knowledge and management of the orotracheal intubation technique in emergency situations, knowledge and management of patients with non-invasive mechanical ventilation therapy, knowledge of the application of the Advanced Life Support algorithm and management of the defibrillator monitor, knowledge, management, and care of patients requiring mechanical restraint, and knowledge and management of patients undergoing fibrinolytic treatment for ischemic stroke. A more comprehensive and detailed description of the training program is available in the publication by Manero et al. (2024) [6].

### 2.6. Evaluation of Competency Development

Upon arrival at the department, all participants completed a self-assessment questionnaire regarding their level of competencies and technical skills specific to emergency nursing in the care of critically ill patients. Additionally, they underwent an external agent competency evaluation using objective rubrics for each of the competencies to be addressed in the mentoring program, prior to the in-person sessions.

After the intervention and the completion of the in-person training sessions, and within a period not exceeding one month, the program participants completed the self-assessment questionnaire again and were re-evaluated by the tutor through an external agent evaluation with objective rubrics [6].

### 2.7. Variables and Measurements Instruments

Demographic variables, such as the sex and age of all participants, were recorded, in addition to previous work experience in hospital emergency departments.

To evaluate both the self-perceived competency level and the competency level resulting from the tutor’s assessment, Patricia Benner’s professional level descriptions with five degrees of competency acquisition were used: novice, advanced beginner, competent, proficient, and expert [23,24]. The measurement instruments, self-assessment questionnaire, and tutor evaluation rubrics were designed and validated by a panel of experts in critical care nursing, achieving content validity indices of 0.877 and 0.9, respectively. A more detailed description of the process is available in the publication by Manero et al. [6]. Thus, for each of the evaluated competencies, two values were obtained: the competency level from the self-assessment questionnaire and the competency level indicated by the external evaluator [6,25].

### 2.8. Data Collection

The recruitment of all participants was carried out by the tutor and the nursing supervisor of the emergency department. All newly recruited personnel who met the inclusion criteria were informed about the existence of this study, its characteristics, and its purpose. If they expressed interest, they were given an informational document with all the details, as well as an informed consent form that they had to read and sign before starting the first objective evaluation conducted in person. Upon agreeing to participate, the tutor sent them an email with a link to complete the initial self-assessment prior to the intervention. All data, both from the self-assessments and the objective evaluations, were collected using questionnaires integrated into the Google Forms application for subsequent analysis.

### 2.9. Data Analysis

Data collection was carried out using the Google Forms application. Subsequently, the data were transferred to Excel 2016 spreadsheet software. All statistical analyses were performed using IBM-SPSS software version 29.0.

The mean of the continuous variable age was calculated, and frequency distributions and proportions were used for the categorical variables sex, previous experience in hospital emergency departments, and competency levels before and after the program obtained through self-assessment and objective evaluation. The comparison between the results of the self-assessment and the objective evaluation, before and after the intervention was performed using the non-parametric Wilcoxon test for paired samples. A difference was considered statistically significant when *p* < 0.05.

### 2.10. Ethical Considerations

This study was conducted in accordance with the Declaration of Helsinki and was approved by the Clinical Research Committee of Aragon (PI 23/2013) for studies involving humans. All individuals who chose to participate received oral information in addition to the informed consent document to be signed.

## 3. Results

### 3.1. Sample Description

Initially, a sample of 69 nurses was recruited. Of these, six ended their contracts before they could begin any study-related activities. The remaining 63 participants completed the tutor evaluation prior to the intervention (TE0), of which 61 completed the corresponding self-assessment questionnaire (SA0).

Therefore, 63 nurses participated in the pre-intervention evaluation phase and received the training intervention. However, after the intervention, three nurses ended their contracts, resulting in a final total of sixty nurses evaluated by the tutor (TE1). Following the exclusion criteria, nurses who did not complete the self-assessment questionnaires in both phases were removed from the self-assessment group. As a result, 52 nurses responded to the self-assessment questionnaire both before (SA0) and after (SA1) the intervention.

**Figure 1:**
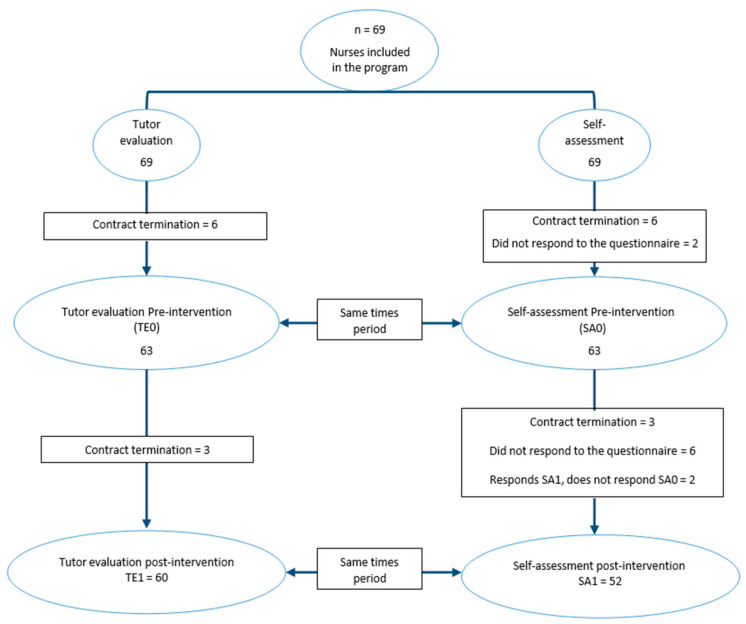
Flowchart of participants in each phase of this study.

Figure 1 shows the flowchart of participants in the different phases of this study.

Of the total participants, 89% (n = 56) were women, with a mean age of 28.9 years, a mode of 24 years, and a median of 29 years. Additionally, 81% (n = 51) had never worked in a hospital emergency department.

### 3.2. Results Obtained in the Evaluations

Before starting the program, the results obtained from the two types of evaluations, self-assessment and external evaluation, across all considered competencies, show a higher percentage of participants at the beginner and advanced beginner competency levels. When comparing the two evaluations, the external evaluation shows higher percentages of nurses classified at these levels than the self-assessment, with a greater number of participants perceiving themselves to have a higher competency level.

In the results of the post-intervention evaluations, there is an increase in the percentage of participants classified at higher competency levels. Additionally, the difference between the percentage values of the self-assessment and the tutor evaluation decreases in three of the evaluated competencies compared to the initial observations. However, this is not the case for the competencies of managing the orotracheal intubation process and managing patients undergoing fibrinolytic treatment for ischemic stroke.

Table 1 shows the percentages obtained in the five competencies evaluated before and after the intervention, both in the self-assessment (SA) and the tutor evaluation (TE), following Benner’s five competency levels.

### 3.3. Comparison Between Self-Assessment (SA) and Tutor Evaluation (TE) Before and After the Intervention

After applying the Wilcoxon test (Table 2), a significant difference (*p* < 0.05) is observed in the results obtained between self-assessment and tutor evaluation at both pre- and post-intervention stages for the competencies of managing the orotracheal intubation process and managing patients undergoing fibrinolytic treatment for ischemic stroke. The self-perceived competency level remains higher than that obtained through external evaluation for the former in both evaluated stages. Conversely, for the latter, participants initially rated themselves higher than the external evaluation before the intervention, but this reversed post-intervention, with participants underrating themselves.

Regarding the remaining competencies, managing patients with non-invasive mechanical ventilation therapy, knowledge of the application of the Advanced Life Support algorithm and defibrillator use, and managing and caring for patients requiring mechanical restraint resulted in significant differences (*p* < 0.05) before the intervention between the two evaluations, with self-assessments rating themselves as more competent. However, this difference (*p* > 0.05) is not observed after the program, with self-assessment and objective evaluation values being very similar (Table 2).

## 4. Discussion

The development of nurses’ competencies is essential to ensure quality care and patient safety in clinical settings. The implementation of a specific training program for nurses in a hospital emergency department, combined with an evaluation approach that includes both self-assessment and tutor assessment, represents a significant step towards improving clinical competencies. This study shows that before the intervention, participants feel more competent than reflected in the tutor’s assessment, highlighting the necessity of a combined evaluation experience to avoid overestimating or underestimating performance.

Studies investigating the agreement/disagreement between self-assessment and assessment by others in nursing competence are scarce, and their findings are inconsistent and contradictory [26]. Clinton et al. (2005) [27] investigated the competence of degree graduates as rated by the graduates themselves and their direct managers in the United Kingdom and found that the ratings were almost compatible. Conversely, other studies reported significant differences between these two ratings; either the self-assessment was higher than that of others, or vice versa [2,13,20,28,29].

Our results revealed discrepancies between self-assessments and tutor evaluations. In our study, it is reflected that in the initial phase prior to the intervention, self-assessments were overestimated compared to the results of the external evaluator, which aligns with what Charles Darwin (1871) noted over a century ago: “ignorance more frequently begets confidence than does knowledge” [29]. This finding is consistent with various studies in which students overestimate their competencies [30,31,32,33]. However, our study has shown that after the intervention, the differences between self-assessment and objective evaluation decrease, indicating that participants have transitioned from the phase of unconscious incompetence to conscious competence. This finding is consistent with Kolb’s experiential learning theory, which postulates that effective learning involves a cycle of concrete experience, reflection, abstract conceptualization, and active experimentation [34,35]. The transition observed in our participants suggests that the intervention not only increased their knowledge and skills but also improved their ability to self-assess and receive constructive feedback.

The results of our study highlight the importance of including self-assessment and tutor evaluation in the development of nursing competencies [33,36]. Self-assessment allows participants to reflect on their own performance, identify strengths and weaknesses, and set personal improvement goals [37,38,39]. This process fosters autonomy and self-efficacy, as demonstrated in previous studies [16]. On the other hand, our results indicate that the greatest competence was acquired after tutor feedback. This finding underscores the importance of the tutor’s role in the educational process, providing expert guidance and specific feedback that helps nurses correct errors and improve their performance [18]. Tutor feedback acts as a positive reinforcement mechanism, consolidating learning and fostering confidence in acquired skills [40,41]. Evaluation from an external perspective can validate or challenge individual perceptions, promoting deeper and more collaborative learning [20,26,27].

Comparing our results with previous studies, we found that self-assessment and external agent evaluation are complementary methods that, when used together, can significantly enhance learning and competency acquisition [42,43]. Various studies have demonstrated that these methods not only improve technical skills but also communication, organizational, and teamwork skills [15,29]. Future studies should consider the inclusion of multiple hospital centers and strategies to improve the longitudinal follow-up of participants to validate and expand these findings.

## 5. Strengths and Limitations

Our study presents several strengths. Firstly, the participants are nurses working in a real-world setting, which contrasts with most published studies that use students as research subjects [22,44]. This characteristic provides greater ecological validity to our findings. Additionally, our study is distinguished by offering prospective data, unlike most existing studies that rely on cross-sectional data [7]. This prospective methodology allows for a better understanding of dynamics and changes over time, providing a more comprehensive and accurate view of the phenomena investigated.

Our study presents some limitations that should be considered. Firstly, the research was conducted in a single hospital, which may limit the generalizability of the results to other hospital settings. Additionally, the hiring system in our country, which often involves short-term contracts, may hinder the long-term follow-up of participants after training. These limitations suggest the need for future studies that include multiple hospital centers and consider strategies to improve the longitudinal follow-up of participants.

## 6. Conclusions

The results obtained highlight the importance of using both self-assessment and tutor evaluation in the training and competency development of nurses. Self-assessment promotes autonomy, but if studies only measure outcomes through nurses’ self-assessments, we might obtain levels that do not align with actual competencies. Tutor evaluation is conducted through a replicable tool with well-defined parameters, making it particularly effective in the learning process. Our study provides evidence that the combination of self-assessment and external evaluation facilitates the transition from unconscious incompetence to conscious competence and also promotes deeper and more sustainable learning.

## Figures and Tables

**Table 1:** Percentages assigned to each competency level before and after the intervention both in the self-assessment (SA0 and SA1) and in the tutor evaluation (TE0 and TE1).

Competency: Knowledge and Management of Patients with Non-Invasive Mechanical Ventilation Therapy.								
	**Before Intervention**				**After Intervention**			
**Competence Level**	**TE0**		**SA0**		**TE1**		**SA1**	
	**Percentage**	**Cumulative Percentage**	**Percentage**	**Cumulative Percentage**	**Percentage**	**Cumulative Percentage**	**Percentage**	**Cumulative Percentage**
Beginner	81.0	81.0	42.6	42.6	0	0	13.0	13.0
Advanced beginner	14.3	95.2	21.3	63.9	23.3	23.3	18.5	31.5
Competent	4.8	100.0	23.0	86.9	63.3	86.7	40.7	72.2
Efficient	0	0	11.5	98.4	13.3	100.0	25.9	98.1
Expert	0	0	1.6	100.0	0	0	1.9	100.0
Competency: Knowledge of the application of the Advanced Life Support algorithm and management of the defibrillator monitor.								
	**Before intervention**				**After intervention**			
**Competence level**	**TE0**		**SA0**		**TE1**		**SA1**	
	**Percentage**	**Cumulative percentage**	**Percentage**	**Cumulative percentage**	**Percentage**	**Cumulative percentage**	**Percentage**	**Cumulative percentage**
Beginner	85.9	85.9	36.9	36.9	10.0	10.0	13.0	13.0
Advanced beginner	6.5	92.4	33.8	70.8	6.7	16.7	29.6	42.6
Competent	7.6	100.0	18.5	89.2	63.3	80.0	31.5	74.1
Efficient	0	0	6.2	95.4	20.0	100.0	24.1	98.1
Expert	0	0	4.6	100.0	0	0	1.9	100.0
Competency: Knowledge, management, and care of patients requiring mechanical restraint.								
	**Before intervention**				**After intervention**			
**Competence level**	**TE0**		**SA0**		**TE1**		**SA1**	
	**Percentage**	**Cumulative percentage**	**Percentage**	**Cumulative percentage**	**Percentage**	**Cumulative percentage**	**Percentage**	**Cumulative percentage**
Beginner	82.5	82.5	44.3	44.3	30.0	30.0	7.4	7.4
Advanced beginner	12.7	95.2	21.3	65.6	18.3	48.3	14.8	22.2
Competent	1.6	96.8	26.2	91.8	3.3	51.7	29.6	51.9
Efficient	3.2	100.0	8.2	100.0	48.3	100.0	40.7	92.6
Expert	0	0	0	0	0	0	7.4	100.0
Competency: Knowledge and management of the orotracheal intubation technique in emergency situations.								
	**Before intervention**				**After intervention**			
**Competence level**	**TE0**		**SA0**		**TE1**		**SA1**	
	**Percentage**	**Cumulative percentage**	**Percentage**	**Cumulative percentage**	**Percentage**	**Cumulative percentage**	**Percentage**	**Cumulative percentage**
Beginner	68.3	68.3	44.3	44.3	4.8	4.8	13.0	13.0
Advanced beginner	30.2	98.4	18.0	62.3	72.6	77.4	16.7	29.6
Competent	0	0	23.0	85.2	12.9	90.3	40.7	70.4
Efficient	1.6	100	13.1	98.4	8.1	98.4	27.8	98.1
Expert	0	0	1.6	100.0	1.6	100.0	1.9	100.0
Competency: Knowledge and management of patients undergoing fibrinolytic treatment for ischemic stroke.								
	**Before intervention**				**After intervention**			
**Competence level**	**TE0**		**SA0**		**TE1**		**SA1**	
	**Percentage**	**Cumulative percentage**	**Percentage**	**Cumulative percentage**	**Percentage**	**Cumulative percentage**	**Percentage**	**Cumulative percentage**
Beginner	65.2	65.2	54.1	54.1	1.7	1.7	20.4	20.4
Advanced beginner	28.3	93.5	27.9	82.0	19.0	20.7	33.3	53.7
Competent	5.4	98.9	8.2	90.2	46.6	67.2	33.3	87.0
Efficient	1.1	100.0	8.2	98.4	31.0	98.3	13.0	100.0
Expert	0	0	1.6	100.0	1.7	100.0	0	0

**Table 2:** Comparison of the average values obtained at the competency level through self-assessment (SA) and tutor evaluation (TE) before and after the intervention.

Competency	Measurement	Paired Sample	Deviation	Dev. Average Error	*p* *
Non-invasive mechanical ventilation therapy	Pre-intervention	E0	0.529	0.068	<0.001
		AE0	1.130	0.145	
	Post-intervention	E1	0.584	0.080	0.846
		AE1	1.017	0.138	
Orotracheal intubation technique in emergency situations	Pre-intervention	E0	0.569	0.073	<0.001
		AE0	1.165	0.149	
	Post-intervention	E1	0.797	0.108	0.002
		AE1	1.022	0.139	
Advanced Life Support algorithm and management of the defibrillator	Pre-intervention	E0	0.606	0.075	<0.001
		AE0	1.108	0.137	
	Post-intervention	E1	0.800	0.109	0.138
		AE1	1.036	0.141	
Fibrinolytic treatment for ischemic stroke	Pre-intervention	E0	0.527	0.068	0.002
		AE0	1.027	0.132	
	Post-intervention	E1	0.793	0.111	<0.001
		AE1	0.983	0.138	
Mechanical restraint	Pre-intervention	E0	0.656	0.084	<0.001
		AE0	1.025	0.131	
	Post-intervention	E1	1.331	0.181	0.15
		AE1	1.049	0.143	

* Wilcoxon signed-rank test for paired samples.

## Data Availability

The dataset is available upon request from the authors.

## Public Involvement Statement

There was no public involvement in any aspect of this research.
